# Native Arteriovenous Fistula Creation in Patients With Continuous Flow Left Ventricular Assist Devices: A Case Report and a Narrative Review

**DOI:** 10.7759/cureus.43751

**Published:** 2023-08-19

**Authors:** Fahmi A Aldhaheri, Samer Koussayer, Bassam Khail, Wesam Abedrabo, Muhammad Ubaid Ullah

**Affiliations:** 1 Nephrology, Dr. Soliman Fakeeh Hospital, Jeddah, SAU; 2 Vascular Surgery, King Faisal Specialist Hospital and Research Centre, Riyadh, SAU; 3 Nursing, King Faisal Specialist Hospital and Research Centre, Riyadh, SAU; 4 Nephrology, King Faisal Specialist Hospital and Research Centre, Riyadh, SAU

**Keywords:** heartmate 3, end-stage kidney disease [eskd], heart failure, hemodialysis, vascular access, arteriovenous fistula [avf], left ventricular assist device [lvad]

## Abstract

Worsening of kidney function after left ventricular assist device (LVAD) implantation is common, and many patients reaching end-stage kidney disease require long-term dialysis. Permanent vascular access in a patient with LVAD remains a clinical dilemma. There is a theoretical concern about the maturation of the arteriovenous fistula in a patient with LVAD due to the absence of a pulsatile flow in these patients. We described a case of successful creation of a left brachial-cephalic AVF in a patient with continuous flow LVAD (Abbott's HeartMate 3TM), which was used for dialysis without issue.

## Introduction

A left ventricular assist device [LVAD] is used as a life-saving modality for patients with end-stage heart failure, which can be utilized as a bridge to cardiac transplantation [BTT] or destination therapy [DT]. It has been shown to improve survival, functional capacity, and quality of life [1].

Worsening of kidney function is common after LVAD implantation. It has been shown that 12.3% of the patients developed acute kidney injury (AKI) in the INTERMACS annual report [2]. A meta-analysis demonstrated that 37% of LVAD patients develop AKI, and 13% require kidney replacement therapy (KRT) [3]. The explanation for AKI is multifactorial. Pigment nephropathy as a result of LVAD-associated hemolysis [4], hemodynamic instability, which leads to further renal ischemia and ends with acute tubular necrosis or the development of right ventricular failure after LVAD implantation, is noticed in approximately 20%-50% of the patients [5-6]. On the other hand, some data showed an improvement in renal function after LVAD implantation, particularly in patients with mild to moderate renal impairment [7,8]. Ideal dialysis access for these patients is still challenging. The dialysis catheter is the most commonly used in LVAD patients, despite the significant infection risk associated with catheters, leading to high morbidity and mortality. Historically, it was thought that in the absence of pulsatile flow in LVAD patients, AVF maturation would be hindered. Due to the higher flow rates associated with AVF, there was also a theoretical concern about worsening hemodynamics leading to high output heart failure. Therefore, arteriovenous grafts were recommended as the preferred AV access in these patients [3].

In the past few years, there have been a few case reports of successful use of AVF in patients with LVAD. Here we report a case of successful AV fistula creation and usage in a patient with LVAD support (Abbott's HeartMate 3TM) who was not maintained on long-term anticoagulation due to bleeding complications.

## Case presentation

A 59-year-old right-hand dominant female was referred for future AV access creation. She has had long-standing type 2 diabetes mellitus, ischemic heart disease with ischemic cardiomyopathy, an ejection fraction of less than 15%, post-implantable cardiac defibrillator (ICD) implantation in 2016, followed by left ventricular assist device (LVAD) implantation with HeartMate 3TM on 17/5/2018 as a bridge for a heart transplant. 

At the time, she was having chronic kidney disease stage 4, mainly secondary to long-standing diabetes and cardio-renal syndrome, with frequent hospitalizations due to decompensated heart failure.

After LVAD implantation, she developed acute kidney injury, mainly due to acute tubular necrosis secondary to frequent hypotension requiring inotropic support and continuous renal replacement therapy via a non-cuffed dialysis catheter. The patient shifted to intermittent hemodialysis as her hemodynamic status improved. Later, a cuffed tunneled dialysis catheter was placed in the right internal jugular vein, and she was kept on intermittent hemodialysis three times a week. Her LVAD speed was set at 5,300 rpms, which provided a flow rate of 3.8 to 4.6 L/min, power consumption of 4.1 to 4.5 watts, and a pulsatility index of 5.3 to 6.1. 

Her course was complicated, with frequent bleeding episodes requiring hospital admissions; initially, she developed lower gastrointestinal bleeding, for which she underwent embolization of two branches originating from the superior rectal artery at the mid- and left-side of the rectum. Later, the patient was admitted multiple times for bleeding from a tracheostomy and was managed conservatively by the otorhinolaryngology team. The cardiovascular team at the time decided to stop warfarin [one year post LVAD implantation] and to keep the patient on an antiplatelet agent. She continued to bleed from the tracheostomy, and aspirin was stopped. There were no more episodes of active bleeding afterwards.

She had recurrent *Candida Glabrata candidemia* that required multiple admissions and treatment with Andilafungin. Infective endocarditis was ruled out by echocardiography. She required multiple removals and re-insertions of a tunneled dialysis catheter after clearance of fungemia. Currently, she is on liposomal amphotericin B after each dialysis session, as recommended by the infectious disease team, as long as the LVAD is in place.

The patient was seen by the interventional nephrology team for ultrasound vessel mapping, which demonstrated the following: brachial artery diameter was 0.31 cm, and the depth was 1.1 cm, the cephalic vein at the elbow was 0.26 cm in diameter and 0.31 cm deep, and the midupper arm diameter was 0.25 cm and the depth was 0.50 cm.

Options were left brachial-cephalic AVF and forearm loop AVG. Given her history of frequent infections, a left brachial-cephalic AV fistula was recommended.

A left brachiocephalic AVF was created on 10th September 2020 under local anesthesia. The artery was pre-operatively marked with ultrasound as no pulse was present, and a standard end-to-side anastomosis was performed. An immediate thrill was detected on clamp release.

**Figure 1:**
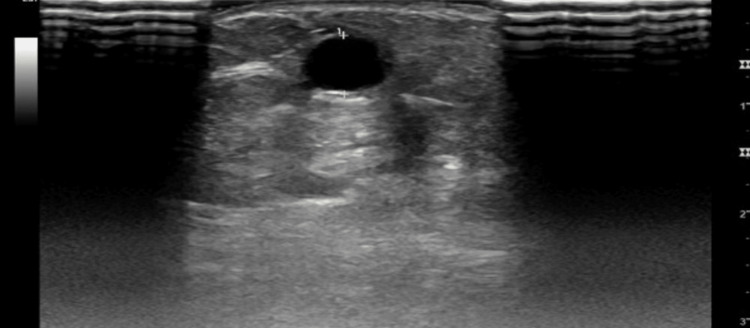
Left brachial-cephalic AVF, 2 cm above the elbow

**Figure 2:**
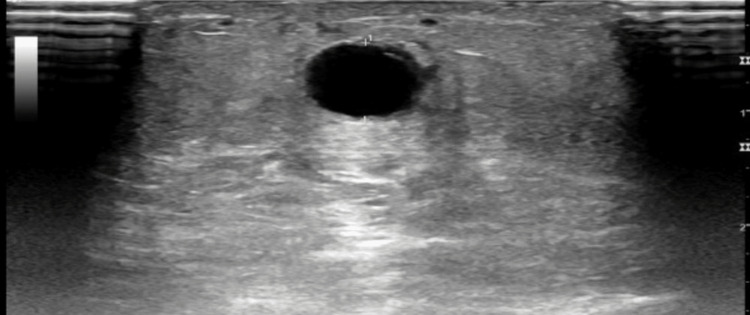
Left brachial-cephalic AVF, 7 cm above the elbow

**Figure 3:**
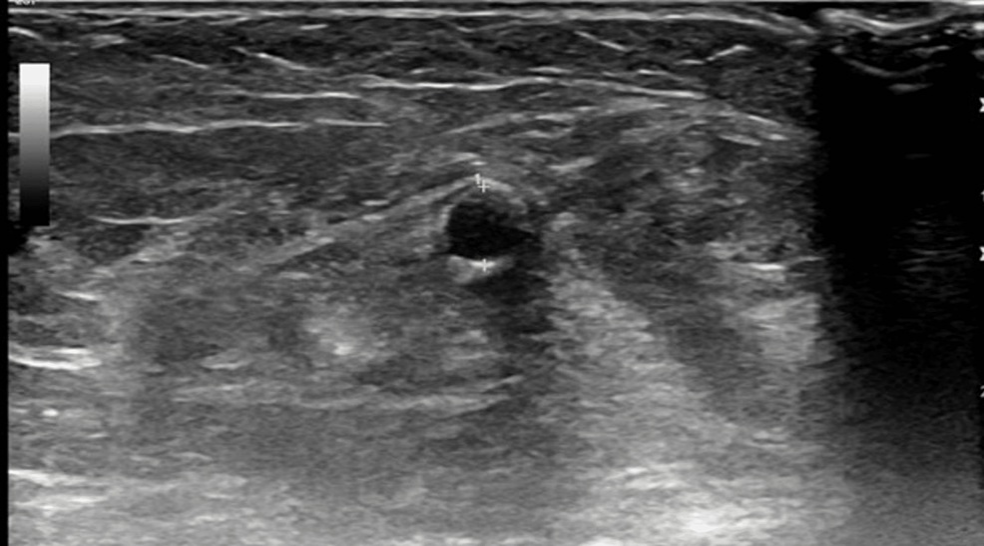
Brachial artery pre-anastomosis

The patient was seen in our clinic for follow-up after six weeks. There was no evidence of dialysis access induced distal ischemia. Ultrasound showed a large accessory cephalic vein. Cephalic vein 2 cm above elbow: diameter was 0.55 cm and depth 0.38 (Figure 1). Cephalic vein 7 cm above elbow: diameter was 0.68 cm and depth 0.45 cm (Figure 2). Brachial artery pre-anastomosis diameter was 0.40 cm (Figure 3).

**Figure 4:**
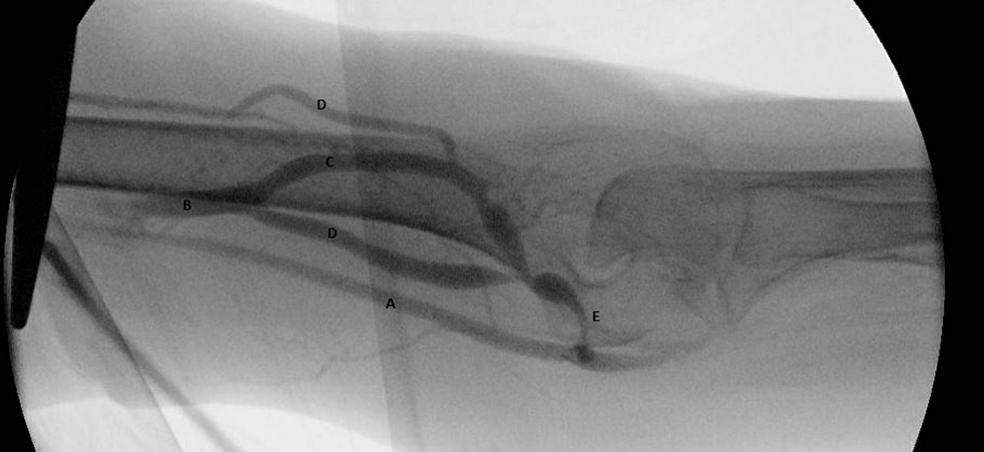
Retrograde fisulogram with the sheath in B. [A: Left brachial artery, B: Left midupper arm cephlaic vein, C: Left cephalic vein-cannulation segment, D: Accessory veins, E: Juxta-anastomosis of the left BC AVF].

**Figure 5:**
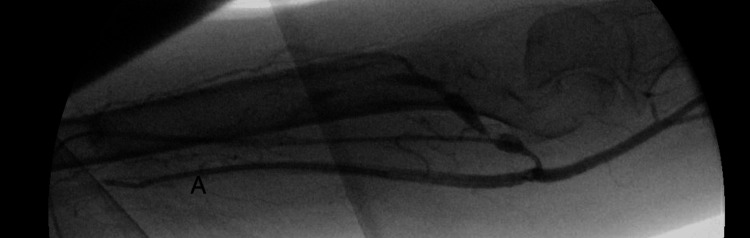
Antergrade arteriogram with the catheter in A

**Figure 6:**
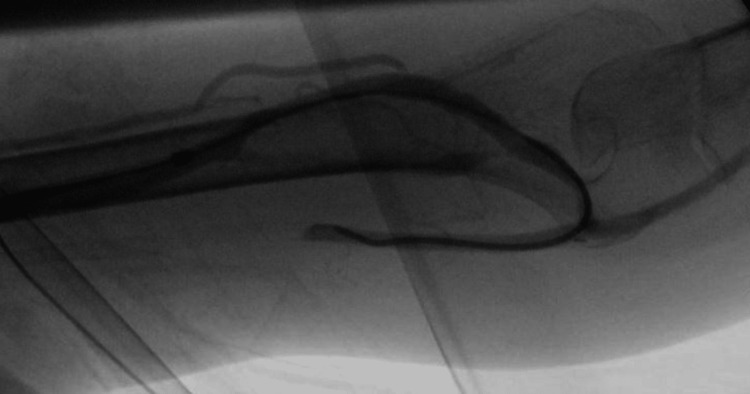
Post-angioplasty antergrade arteriogram with the catheter in A

On 24th Nov 2020, ultrasound assessment before cannulation showed the diameter of the fistula body was 0.68 cm and the depth was 0.45 cm. The fistula was cannulated according to our institutional initial dialysis cannulation protocol using 17G needles, followed by 16G and 15G needles after weekly intervals. Assessment of AVF flow was done after three months using ultrasound dilution (Transonic, Inc.), which showed a rate of 410 mL/min and 0% recirculation. The fistula was continued to be cannulated using a 15-gauge needle with a pump flow rate of 350-400 ml/min and acceptable arterial and venous pressures. Access flow was measured at six months and showed a rate of 170 ml/min and 74% recirculation. The patient was booked for angiography, which showed severe stenosis in the distal part of the fistula, juxta-anastomosis, and arterial anastomosis. Less than 50% stenosis was seen within the left subclavian vein. Angioplasty was done using a 4 x 40 mm angioplasty balloon catheter in the anastomosis and juxta-anastomosis, and a 6 x 40 mm balloon was used in the body of the fistula. A follow-up angiogram showed some residual stenosis in the inflow segment. A 5 x 60 mm drug-coated balloon was then used to angioplasty the arterial anastomosis and the juxta-anastomosis, with no significant residual stenosis after that (Figures 4-6).

## Discussion

LVAD is a mechanical pump that augments cardiac output. It consists of an inflow cannula implanted at the left ventricular apex and an outflow cannula that is connected to the ascending aorta. A pump draws blood from the left ventricle, offloads it, and thrusts the blood into the ascending aorta. A system controller displays the LVAD parameters and helps adjust the device settings. The pump connects to the controller and power supply (batteries) via a driveline.

Since DeBakey and colleagues implanted the first pneumatically driven LVAD in 1966 [9], the technology has evolved significantly, especially in the last two decades. These devices are divided into first, second, and third generations based on the underlying mechanics. The first generation of LVADs used a pulsatile flow pump design that was driven by either pneumatic or electrical drive systems. It was approved for use as a bridge to cardiac transplantation in 1994. However, now-obsolete first-generation LVADs had several disadvantages: larger size, higher risk of infection, and malfunction. The current second- and third-generation devices use continuous flow. Continuous flow pumps have several advantages, viz., smaller size, decreased susceptibility to infections, less noise, less motion, and less surgical dissection to implant the pump [10]. Patients dependent on these devices do require anticoagulation with target INR 2-3 and antiplatelets (Aspirin at least 81mg) to prevent pump thrombosis.

The second generation (Abbott's HeartMate IITM) is an axial continuous-flow circulatory pump, while the third generation LVADs are (HeartMate 3 and HeartWare HVAD) centrifugal continuous-flow circulatory pumps. HeartMate III employs a magnetically levitated pump and generates an artificial pulse by altering the speed of the pump every 2 seconds to minimize stasis of blood in the rotor and decrease the risk of thrombosis and hemolysis [11]. It is inserted in the pericardium, which could help in maintaining the peritoneal membrane integrity for consideration of peritoneal dialysis in the future. HM III was approved by the FDA in 2017 as a bridge to transplant and in 2018 as a destination therapy. Mehra et al. reported better outcomes at six months with centrifugal continuous flow devices due to the lower rate of reoperation for pump dysfunction compared to axial flow pumps [12].

As discussed, some patients will need to continue on long-term dialysis after LVAD implantation. Continuation of hemodialysis via central venous catheters is associated with increasing the risk of infection, especially in the presence of other intravascular devices. Initial expert opinion used to be in favor of the AVGs due to their theoretical advantages, as discussed before. However, successful AVF creation and usage in patients with second-generation LVADs have been reported. There have been 10 cases published so far with successful AVF use in LVAD patients, all with HeartMate II devices. To our knowledge, this is the first case being reported with an AVF created and used on a patient with LVAD HeartMate III. Another unique feature of our case is the lack of anticoagulation or platelet inhibition that is normally required in patients with LVAD and may be inferred as a confounder when looking at the patency of the AV accesses.

Sasson et al. reported two cases of radial cephalic arteriovenous fistulae that required assisted maturation for long-term patency and were used for 329 (until patient death) and 511 days [13]. A case report was published by Chin et al. of radial artery to basilic vein AVF, which was transposed and superficialized and was subsequently used successfully [14]. A case series by Schaefers and Ertmer reported the creation of AVF in three cases that did not require assisted maturation. The AVF in the first case remained patent for 350 days; the second AVF was used for 337 days until the patient died; and the third AVF was used for 116 days after creation, then required surgical revision for thrombosis and was used for four months until the patient died [15]. Calenda et al. described successful hemodialysis in three patients with AVF [16]. One died because of LVAD failure, and the other was complicated by a large access hematoma for which a tunneled dialysis catheter was placed for dialysis. The third one was free of any complications or further intervention. Khawaja et al. described a successful case of brachial-cephalic AVF used for dialysis without further intervention [17].

The primary failure rate of AVF in the non-LVAD population has been reported in the range of 18% to 60% [18] and should likewise be considered one of the limitations of AVF in patients with LVAD. The previously assumed theoretical concerns related to AVF in this patient population do not seem to be backed by evidence. On the contrary, associations between AVGs and a higher incidence of infections and thrombosis are more established. Given the growing number of reported cases of successful and long-term AVF use, the choice of AV access should take patient-specific factors into consideration more so than device-specific factors. 

Peritoneal dialysis [PD] can be considered an alternative modality, especially with the third-generation LVAD that spares the peritoneal membrane. PD is associated with less infection compared to a hemodialysis catheter [19]. In addition, it removes fluid more gently, providing more hemodynamic stability. There are few cases reported that show the feasibility of PD in LVAD patients [20].

## Conclusions

AVF can be created and used successfully in LVAD patients. Concerns about maturation are not proven. The main purpose of dialysis access is to provide efficient dialysis, minimizing interventions and complications. The decision to secure optimal AV access in patients with LVAD should follow the ESKD Life Plan as recommended by the recent KDOQI guidelines.
